# A Nomogram to Predict Survival in Patients With Locoregional Recurrent Nasopharyngeal Carcinoma Receiving Comprehensive Treatment

**DOI:** 10.3389/fonc.2022.892510

**Published:** 2022-06-16

**Authors:** Ying-Hong Wei, Ying Wang, He Li, Chi-jie Wang, Song-Ran Liu, Zi-Lu Huang, Guan-Nan Wang, Ya-Lan Tao, Yun-Fei Xia

**Affiliations:** ^1^State Key Laboratory of Oncology in South China, Collaborative Innovation Center for Cancer Medicine, Guangdong Key Laboratory of Nasopharyngeal Carcinoma Diagnosis and Therapy, Sun Yat-sen University Cancer Center, Guangzhou, China; ^2^Department of Radiation Oncology, Sun Yat-sen University Cancer Center, Guangzhou, China; ^3^Medical Statistics, School of Public Health, Sun Yat-sen University, Guangzhou, China; ^4^Department of Pathology, Sun Yat-sen University Cancer Center, Guangzhou, China; ^5^Department of Head & Neck Surgery, Sun Yat-sen University Cancer Center, Guangzhou, China

**Keywords:** recurrent nasopharyngeal carcinoma, prognostic stratification, nomogram, inflammation-nutritional markers, comprehensive treatment

## Abstract

**Objective:**

This study aimed to establish a prognostic stratified model of chemotherapy-based comprehensive treatment for patients with locoregional recurrent nasopharyngeal carcinoma (lrNPC), to help individualized treatment decision-making.

**Materials and Methods:**

This study retrospectively reviewed patients with lrNPC who received chemotherapy-based comprehensive treatment from January 1, 2010, to December 31, 2018. A total of 422 eligible patients were divided into test (n = 338) and validation (n = 84) cohorts. A LASSO cox regression model was used to identify significant prognostic factors for overall survival (OS) in the test cohort. A nomogram was then developed based on a combined consideration of clinically meaningful prognostic factors and statistically significant prognostic factors. The performance of the nomogram was assessed with Harrell’s concordance index (C-index) and calibration plots.

**Results:**

Five significant factors were identified: age, albumin (ALB), T stage after recurrent (rT), neutrophil to lymphocyte ratio (NLR), and systematic immune-inflammation index (SII). The nomogram was established with these five factors. C-index was 0.636 in the test cohort and 0.610 in the validation cohort. The calibration curves for the OS rate at 3, and 5 years showed an excellent agreement in both cohorts. In addition, the corresponding risk classification system successfully classified patients into low- and high-risk groups and performed well in stratification (P < 0.001).

**Conclusions:**

The nomogram shows well prognostic performance for lrNPC patients receiving chemotherapy-based comprehensive treatment.

## Introduction

Nasopharyngeal carcinoma (NPC) is one of the most common malignant cancers in the east and southeast Asia and is highly sensitive to radiation (1, 2). However, about 10%-15% of patients experience locoregional recurrence after primary treatment (3, 4). For patients with locoregional recurrent nasopharyngeal carcinoma without distant metastasis (lrNPC), treatment strategies are mainly divided into three types: surgery, re-irradiation, and chemotherapy-based comprehensive treatment. Surgery and re-irradiation are limited due to the initial treatment and complex structure of the nasopharynx (5, 6), so chemotherapy-based comprehensive treatment, is a better choice for lrNPC and confers survival benefits by increasing the local control rate and eradicating micrometastases (7).

Accurate prognostic stratification is critical for therapeutic decisions making. Most studies constructed the prognostic stratification models in lrNPC mainly focused on patients treated with surgery or re-irradiation after recurrence, there are few studies on the prognosis prediction of chemotherapy-based comprehensive treatment (8, 9). In addition, doctors make chemotherapy-based comprehensive treatment strategy were based on individualized judgement and patient characteristics, which is short of quantifiable criteria. Therefore, it is necessary to find good prognostic indicators and establish a prognostic stratification model for chemotherapy-based comprehensive treatment.

Inflammation is considered to be cancer-initiating factors and participate in the entire process of cancer development (10). NPC is a well-recognized inflammatory cancer, adding inflammatory factors to the prognostic stratification system can help to improve the sensitivity of prognostic stratification. Among the established cancers, there is increasing evidence that cancer-related inflammation, tumor stage, and clinical condition jointly affect the prognosis of patients (11). The general nutritional condition of the patients with recurrence is worse than before, and many patients are even difficult to receive anti-recurrent treatment due to poor nutritional condition, so the nutritional indicators are critical for lrNPC patients. However, most of these markers were evaluated separately, so we collected as many prognostic biomarkers as possible, assessed the value of these biomarkers, and chose the optimal ones to construct a prognostic stratification model, aiming to refine patient stratification conveniently and efficiently.

## Materials and Methods

### Study Cohort

We retrospectively reviewed a consecutive cohort of 840 patients with histologically confirmed NPC who recurrent after curative radiotherapy and had been treated at the Sun Yat-sen University Cancer Center from January 2010 through December 2018. Patients who met the following criteria were enrolled in the study: histologically confirmed lrNPC; had received chemotherapy-based comprehensive treatment. Patients were excluded if they were in the following situation: had tumor metastasis or other malignant tumors; a surgical alone treatment; a re-irradiation alone treatment; a chemotherapy alone treatment; incomplete information. The study was approved by the Institutional Review Board of Sun Yat-sen University Cancer Center (B2021-057-01), and informed consent was waived.

### Restaging After Recurrence

Imaging data of patients, including but not limited to, MRI of the nasopharynx and neck, X-ray or CT of chest, ultrasound or CT of the abdomen, whole-body bone scan or whole-body PET-CT were collected at the time of diagnosis of recurrence. MRI images were independently evaluated by two radiologists, and then restaging according to the criteria of the eighth edition of the tumor-node-metastasis (TNM) stage system by two clinicians specializing in nasopharyngeal carcinoma. Any differences were resolved by consensus.

### Treatment

For patients with lrNPC, there are many options recommended by NCCN guideline for chemotherapy-based comprehensive treatment, the choice of chemotherapy regimen was administered at the physician’s judgment and the patient’s consent. In the study cohort, chemotherapy regimens had been collected as follows: docetaxel plus cisplatin plus 5-fluorouracil (TPF), docetaxel plus cisplatin (TP), gemcitabine plus cisplatin (GP), and cisplatin plus 5-fluorouracil (PF). 21 days a cycle. Radiotherapy, molecular-targeted therapy, and immune checkpoint therapy were permitted in the cohort.

### Data Collection

Demographic, clinical, and laboratory data before receiving chemotherapy were collected. Demographic data included age at recurrence, sex, and smoke state. Clinical data included TNM category after recurrence (rTNM), body mass index (BMI), and over survival (OS). OS was measured from the date of diagnosis for recurrence to the date of death or last follow-up (December 31, 2020). Laboratory data including white blood cells (WBC), lymphocyte (LYM), neutrophils (NEU), hemoglobin (HGB), platelets (PLT), monocytes (MONO), lactate dehydrogenase (LDH), and albumin (ALB) were collected. And neutrophil to lymphocyte ratio (NLR), platelet to lymphocyte ratio (PLR), lymphocyte to monocyte ratio (LMR), systemic inflammatory response index (SIRI), systematic immune-inflammation index (SII), prognostic nutritional index (PNI), and advanced lung cancer inflammation index (ALI) had been calculated as follows:


NLR = neutrophils/lymphocytes;

PLR = platelets/lymphocytes;

LMR = lymphocytes / monocyte;

SIRI=neutrophils×monocytes/lymphocytes;

SII = neutrophils × platelets/lymphocytes;

PNI = Albumin+5×lymphocytes;

### Statistical Analysis

The whole cohort was randomly divided into test set and validation set in a proportion of 8:2. Demographic, clinical, and laboratory data of all patients in the test cohort were analyzed for association with OS by using LASSO cox regression. The variables for the selected minimum lambda chosen by fivefold cross-validation were included in the multivariable model for building of the nomogram. The predictive accuracy of the nomogram was assessed by Harrell’s concordance index (C-index) and calibration plots in both the test and the validation set. Overall survival rates were calculated using the Kaplan-Meier method, and the log-rank test was used to perform comparisons between groups. HRs and 95% CIs were calculated with an unstratified Cox proportional hazards model. The cut-off values of LDH, WBC, LYM, NEU, HGB, PLT, MONO, and ALB were based on our hospital’s test threshold, which was based on “CLSI Defining, Establishing and Verifying Reference Ranges in the Clinical Laboratory”, the study of Ichihara K on derivation of reference intervals (12), and quality control requirements of our hospital laboratory equipment. The cut-off value of BMI was from the study of Krishnan Bhaskaran on 3.6 million adults (13), the cut-off values of age, NLR, PLR, LMR, SIRI, SII, PNI, and ALI were calculated for over survival by X-tile (14), the cut-off values of points were calculated by the median value. Two-tailed P-values < 0.05 were considered statistically significant. All statistical analyses were performed using SPSS 25.0 (Chicago, IL, USA) and R Studio v1.4.1106.

## Results

### Patient Characteristics and Survival

Between Jan 1, 2010, and Dec 31, 2018, a total of 422 patients met the criterion were identified in the study. The male (n = 328)-to-female (n = 94) ratio was 3.5:1. The median age was 46.5 years and most of the patients were non-smokers.

According to TNM stage system, 157 (37.2%) patients were rT0 to rT2, 135 (32.0%) were rT3, 130 (30.8%) were rT4. For the N stage, 219 (51.9%) patients had rN negative (rN0), 203 (48.1%) patients had rN positive (rN1-rN3). All patients received chemotherapy-based comprehensive treatment, 118 patients receiving GP, 97 patients receiving PF, 123 patients receiving TP, 84 patients receiving TPF. All patients received radiotherapy, 118 patients received targeted therapy (69 received Nitozumab, 3 received Apatinib, 12 received Cetuximab, 9 received Avastin, 25 received Endostatin), 8 patients received immunotherapy (2 received atezolizumab,1 received pembrolizumab, 5 received toripalimab). This was showed in **Table S1**. The difference in survival among the different treatment groups was shown in **Figure S1**. 338 patients were assigned to the test set and the remaining 84 patients were assigned to the validation set. Detailed clinical characteristics of patients from the test and validation sets were summarized in **Table 1**. In the test group, the median follow-up was 4.21 (95%CI, 3.64-4.78) years, while the median OS was 3.78 (95%CI, 3.18-4.39) years, the 5-year OS rate was 39.9%, a total of 167 (49.7%) patients died at the end of the follow-up. In the validation group, the median follow-up was 4.62 (95%CI, 3.07-6.17) years, while the median OS was 3.36 (95%CI, 2.93-3.79) years, the 5-year OS rate was 27.7%, a total of 47 (56%) patients died at the end of the follow-up.

**Table 1:** Baseline characteristics of patients.

Characteristic	Patients, No (%)		
	Total	Test set	Validation set
	(N=422)	(N=338)	(N=84)
Sex			
Male	328 (77.7)	257 (76.0)	71 (84.5)
Female	94 (22.3)	81 (24.0)	13 (15.5)
Age (years)			
≤55	341 (80.8)	275 (81.4)	66 (78.6)
>55	81 (19.2)	63 (18.6)	18 (21.4)
Smoker			
Yes	327 (77.5)	266 (78.7)	61 (72.6)
No	95 (22.5)	72 (21.3)	23 (27.4)
rT classification^a^			
rT0-rT2	157 (37.2)	117 (34.6)	40 (47.6)
rT3	135 (32.0)	111 (32.8)	24 (28.6)
rT4	130 (30.8)	110 (32.6)	20 (23.8)
rN classification^a^			
rN0	219 (51.9)	180 (53.3)	39 (46.4)
rN1-rN3	203 (48.1)	158 (46.7)	45 (53.6)
BMI (Kg/m^2^)			
≤21	147 (34.8)	120 (35.5)	27 (32.1)
>21	275 (65.2)	218 (64.5)	57 (67.9)
ALB (g/L)			
≤250	65 (15.4)	51 (15.1)	14 (16.7)
>250	357 (84.6)	287 (84.9)	70 (83.3)
LDH (U/L)			
≤250	402 (95.3)	320 (94.7)	82 (97.6)
>250	20 (4.7)	18 (5.3)	2 (2.4)
WBC (10^9^/L)			
≤9.5	394 (93.4)	315 (93.2)	79 (94.0)
>9.5	28 (6.6)	23 (6.8)	5 (6.0)
LYM (10^9^/L)			
≤1.1	191 (45.3)	151 (44.7)	40 (47.6)
>1.1	231 (54.7)	187 (55.3)	44 (52.4)
NEU (10^9^/L)			
≤6.3	378 (89.6)	302 (89.3)	76 (90.5)
>6.3	44 (10.4)	36 (10.7)	8 (9.5)
HGB (10^9^/L)			
≤130	146 (34.6)	119 (35.2)	27 (32.1)
>130	276 (65.4)	219 (64.8)	57 (67.9)
PLT (10^9^/L)			
≤100	3 (0.7)	3 (0.9)	0 (0.00)
>100	419 (99.3)	335 (99.1)	84 (100.00)
MONO (10^9^/L)			
≤0.6	385 (91.2)	306 (90.5)	79 (94.0)
>0.6	37 (8.8)	32 (9.5)	5 (6.0)
PLR			
≤307.14	351 (83.2)	281 (83.1)	70 (83.3)
>307.14	71 (16.8)	57 (16.9)	14 (16.7)
LMR			
≤2.28	110 (26.1)	89 (26.3)	21 (25.0)
>2.28	312 (73.9)	249 (73.7)	63 (75.0)
NLR			
≤2.75	144 (34.1)	116 (34.3)	28 (33.3)
>2.75	278 (65.9)	222 (65.7)	56 (66.7)
SIRI			
≤1.96	320 (75.8)	253 (74.9)	67 (79.8)
>1.96	102 (24.2)	85 (25.1)	17 (20.2)
SII			
≤578.85	140 (33.2)	112 (33.1)	28 (33.3)
>578.85	282 (66.8)	226 (66.9)	56 (66.7)
PNI			
≤50.45	219 (51.9)	177 (52.4)	42 (50.0)
>50.45	203 (48.1)	161 (47.6)	42 (50.0)
ALI			
≤341.57	265 (62.8)	214 (63.3)	51 (60.7)
>341.57	157 (37.2)	124 (36.7)	33 (39.3)

a According to the 8th edition of the AJCC/UICC stage system. rT0–rT2 and rN1-rN3 were grouped together due to the small number of patients with those stage.

BMI, body mass index; ALB, albumin; LDH, lactate dehydrogenase; WBC, white blood cells; LYM, lymphocyte; NEU, neutrophils; HGB, hemoglobin; PLT, platelets; MONO, monocytes; PLR, platelet to lymphocyte ratio; LMR, lymphocyte to monocyte ratio. NLR, neutrophil to lymphocyte ratio; SIRI, systemic inflammatory response index; SII, systematic immune-inflammation index; PNI, prognostic nutritional index, ALI, advanced lung cancer inflammation index.

### Independent Prognostic Factors Associated With OS

**Figure 1:**
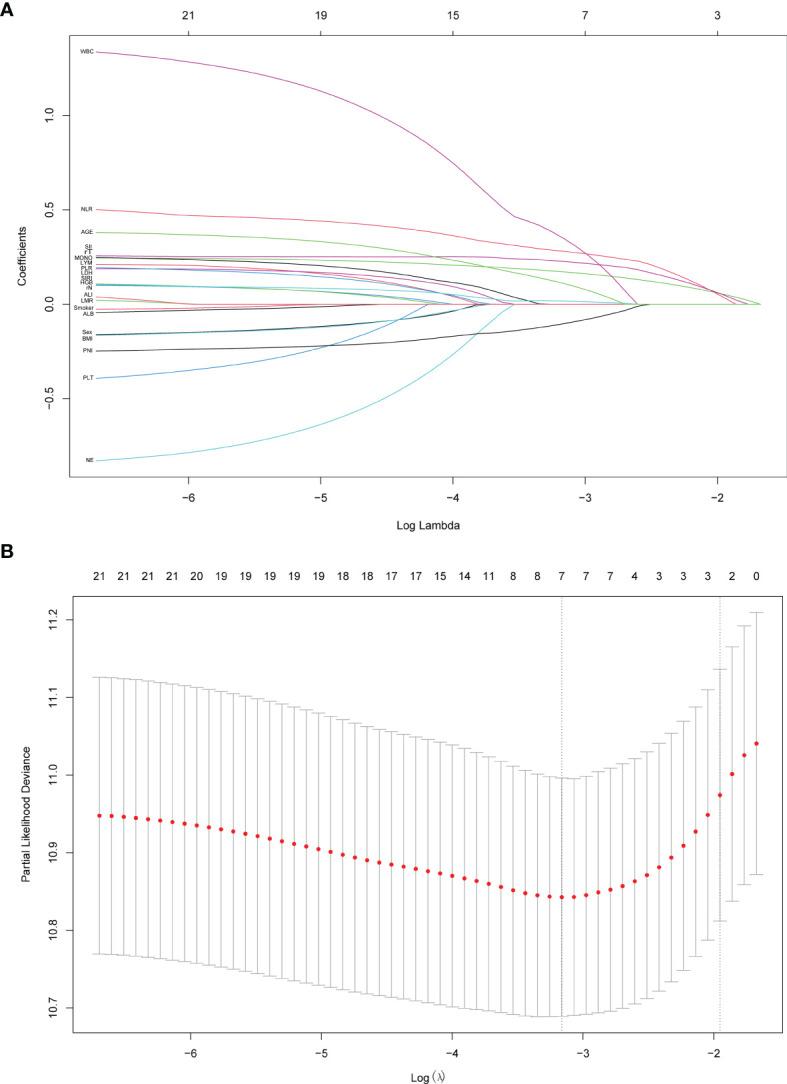
Predictor variables selection using the least absolute shrinkage and selection operator (LASSO) binary cox regression model. **(A)** LASSO coefficient profiles of the 21 predictor variables. **(B)** Tuning parameter λ selection in the LASSO model used fivefold cross-validation. Dotted vertical lines were drawn at the optimal values by using the minimum criteria (λ.min) and the 1 standard error of the minimum criteria (the 1-SE criteria, λ.1se). λ.1se value of 0.142 was chosen and screened out three optimal predictors. The figures were created using R Studio software v1.4.1106.

21 variables listed in **Table 1** were included in the LASSO cox regression for their association with OS in the test group (**Figure 1**), and it indicated the following prognostic factors: rT, NLR, and SII were independent risk factors. The coefficients for these features were calculated and shown in **Figure 1A**. The most prognostic covariates were selected by the lambda value within one standard error from the minimum (λ.1se) to predict OS by fivefold cross-validation as presented in **Figure 1B**.

### Construction and Assessment of the Nomogram Model

**Figure 2:**
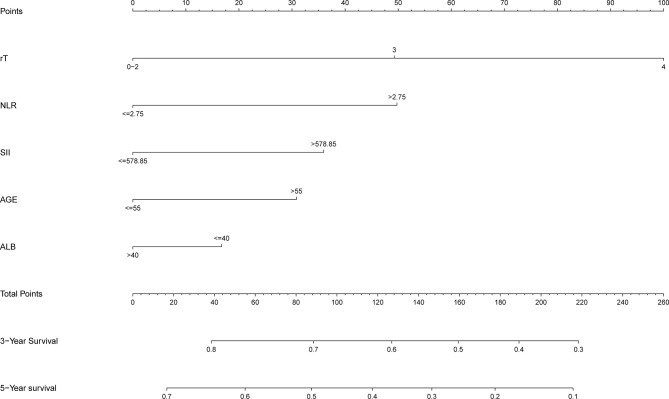
Nomogram to predict 3- and 5-year OS rates of lnNPC patients. rT, T stage after recurrence; NLR, neutrophil to lymphocyte ratio; SII, systematic immune-inflammation index; ALB, albumin.

**Figure 3:**
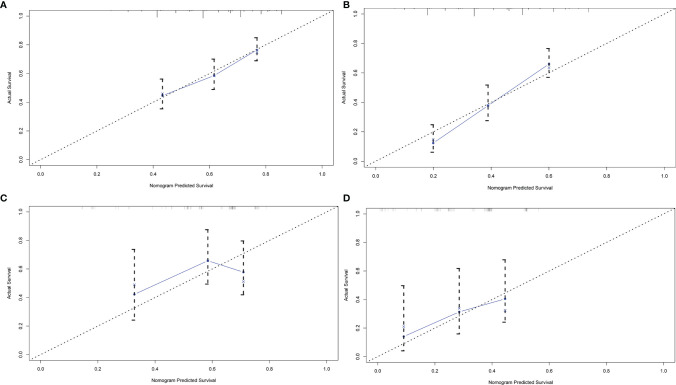
The calibration curve of nomogram for predicting OS. **(A)** three years in the test group **(B)** five years in the test group **(C)** three years in the validation group **(D)** five years in the validation group. Nomogram-predicted OS is plotted on the x-axis; actual OS is plotted on the y-axis.

In order to better reflect the clinical prognostic value, clinically meaningful prognostic factors were also included in the construction. Taking clinical meaningful factor age and ALB into account, a nomogram with 5 independent prognostic factors that could predict the 3-, and 5-year OS was developed in the test cohort (**Figure 2**), and the predictive ability of this model was assessed by C-index, which was 0.636 in the test cohort and 0.610in the validation cohort. The calibration plots adjusted by bootstrapping with 1,000 samples were used to evaluate the performance of the nomogram graphically (**Figure 3**). The calibration curves for the OS rate at 3, and 5 years in the test cohort and the validation cohort overlapped well with reference lines demonstrating excellent performance of the nomogram.

### Prognostic Risk Stratification

**Figure 4:**
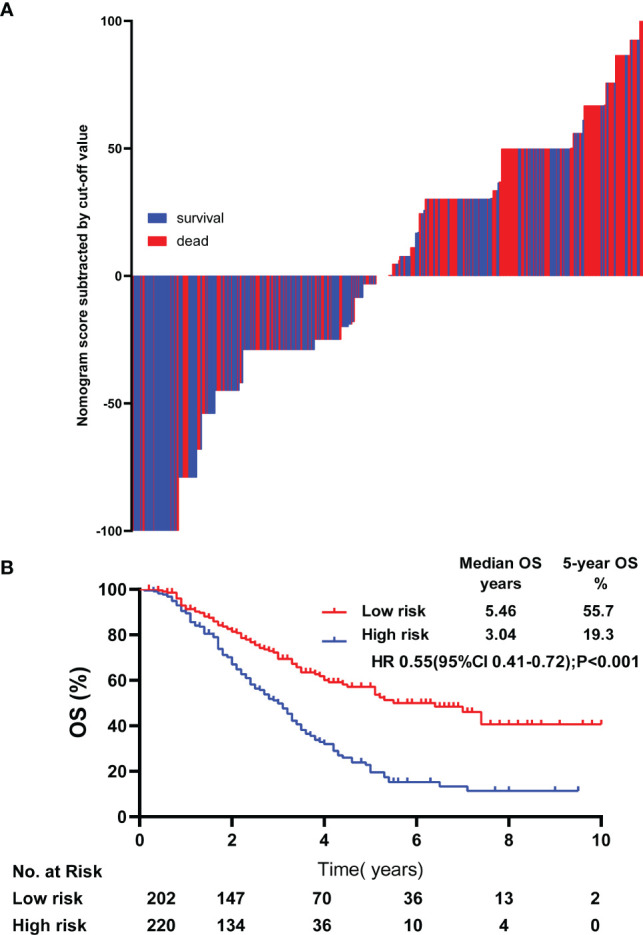
Risk stratification of lrNPC patients. The calculated risk scores for each patient in the whole cohort **(A)**. Kaplan-Meier survival curves of OS in the whole cohort **(B)** OS, over survival; HR, Hazard Ratio.

By using the nomogram established, the detailed score of the 422patients was calculated. The median score was 52.05 and the difference between each patient’s score and the median was shown in **Figure 4A**. Using the median score as the cutoff value, all patients were divided into high-risk groups and low-risk groups. Patients in high-risk group (≥ 52.05) had worse overall survival in comparison with the patients in low-risk group (< 52.05), (P < 0.001, HR: 0.55,95% CI: 0.41-0.72, **Figure 4B**).

## Discussion

In this study, we established a prognostic stratified model for lrNPC to help oncologists make treatment decisions. By applying this model, high-risk patients could increase the treatment intensity, and low-risk patients could reduce the treatment intensity, effectively avoiding over-treatment and under-treatment.

Currently, therapeutic decisions making and prognostic stratification for NPC are based on TNM stage system, and patients are stratified according to the anatomical region. Instead of full consideration of the biological heterogeneity of cancer (15), this is often insufficient for prognostic stratification and individualized treatment. In addition, the stage system of recurrence uses the TNM stage, which is the same system for initial diagnosed NPC, and this would decrease the accuracy of the prognostic stratification (16, 17).For recurrent patients, accurate prognostic stratification and individualized treatment are very important (18, 19). The structure of the nasopharynx has changed after primary treatment, as well as the receptivity to anti-recurrence treatment. This increased the difficulty for clinicians to choose effective treatment measures, and clinicians need to consider the efficacy as well as side effects more carefully. In our study, prognostic factors were identified based on analysis of patients with lrNPC, and individual prognostic risk scores were calculated for risk stratification. The prediction of OS in low-risk group patients was significantly better than that in high-risk group patients, suggesting that our model performed well in prognostic stratification of patients with lrNPC.

Cancer biology is constantly shifting from a “cancer cell-centric” view to a broader concept that places inflammation as a cancer biomarker, the key role of inflammation in carcinogenic process has attracted attention (20), and inflammation is a recognized hallmark of cancer, which greatly promotes the development of cancer (21). Inflammation can affect both the treatment response and prognosis in caners (22–25), and this had been confirmed in many clinical studies of different cancer types (26–32).The study of Leggas M in patients with non-small cell lung cancer proved that intensive anti-inflammatory therapy can improve the efficacy of chemotherapy drugs, reduce blood toxicity, and change the pharmacokinetics of drugs (33). In the past few years, a number of systemic inflammation markers have been used as prognostic predictors, and there are also a lot of studies that have confirmed the value of these indicators in predicting the prognosis of different solid tumors (34–37). Considering the accessibility, ease of use, and economic cost of these markers, 15 common blood-related markers were selected in our study, and through further screening, 2 blood-related prognostic factors were selected to construct the nomogram.

ALB was also added as a prognostic factor in constructing the nomogram in our study. According to the latest studies, ALB is strongly associated with all-cause mortality, including cancer, cardiovascular diseases, and respiratory diseases, and lower ALB is associated with an increased risk of death (38–42). This is further confirmed in all cancer types, The studies in cervical carcinoma and vulvar squamous cell carcinoma had proved the value of nutritional indicators as independent risk factors (31, 43). And ALB reduction has been independently shown to be closely related to precachexia/cachexia body composition, which indicated a worse prognosis and higher mortality (44–46). The results of our study were therefore consistent with previous research conclusions.

This study has several advantages. Firstly, most of the current studies only focus on partial inflammation markers, in our study, we had conducted a systematic and comprehensive analysis of all markers which have been proven to have predictive value in various studies, and screened out the factors combined with the highest predictive power to help clinical decision-making. Secondly, for the risk stratification of patients with recurrence, You R and his colleague established a special stratification system for operable patients (9). For inoperable patients, chemotherapy-based comprehensive treatment is the main treatment option but is short of specialized stratification systems. To the best of our knowledge, this study collected the largest number of patients who have been treated with chemotherapy-based comprehensive treatment after recurrence. Additionally, the markers selected in this study can be obtained from routine clinical examinations, which do not increase the economic burden on patients. At the same time, these markers have high accessibility and universality, and can be routinely available in any hospital.

Besides these strengths, this study has some limitations: firstly, as a retrospective study, we could not collect all information, such as Epstein-Barr Virus DNA or C-reactive protein (47, 48), whether there is a better factor combination in predicting OS remains to be ascertained. Secondly, all the data came from our institution, so sample bias may exist. However, it should not influence the results, as the cohort consisted of large sample size and all eligible patients were consecutively enrolled.

## Conclusions

In conclusion, we established a prognostic stratification model for lrNPC patients who received chemotherapy-based comprehensive treatment, and we hope that our work can help clinicians develop individualized and accurate treatment strategies.

## Data Availability Statement

The raw data supporting the conclusions of this article will be made available by the authors, without undue reservation.

## Ethics Statement

The studies involving human participants were reviewed and approved by Institutional Review Board of Sun Yat-sen University Cancer Center. Written informed consent for participation was not required for this study in accordance with the national legislation and the institutional requirements.

## Author Contributions

Study concept: YW,YT,Y-FX. Data acquisition: All authors. Statistical analysis and writing of manuscript: YW, YW. Supervision: Y-FX, YT. Manuscript review: All authors. All authors listed have made a substantial, direct, and intellectual contribution to the work and approved it for publication.

## Funding

The study was supported by the Guangdong Hong-Kong Technology Cooperation Funding Science (No. 2021A0505110010), National Natural Science Foundation of China (No. 82103437), and Chinese Postdoctoral Science Foundation (No. 2021M693649).

## Conflict of Interest

The authors declare that the research was conducted in the absence of any commercial or financial relationships that could be construed as a potential conflict of interest.

## Publisher’s Note

All claims expressed in this article are solely those of the authors and do not necessarily represent those of their affiliated organizations, or those of the publisher, the editors and the reviewers. Any product that may be evaluated in this article, or claim that may be made by its manufacturer, is not guaranteed or endorsed by the publisher.

## Supplementary Material

Supplementary Figure 1 Over survival in different treatment groups. **(A)** Difference in survival among populations with different radiotherapy conditions. Radiotherapy concurrent with chemotherapy vs. radiotherapy plus chemotherapy, p=0.910 **(B)** Difference in survival among populations with different chemotherapy regimens GP vs. TP, p=0.436; GP vs. TPF, p=0.905; GP vs. PF, p=0.331 **(C)** Difference in survival among populations with different targeted therapy conditions. received targeted therapy vs. without received targeted therapy, p=0.230; **(D)** Difference in survival among populations with different immunotherapy conditions. received immunotherapy vs. without received immunotherapy, p=0.196; 2-tailed α risk is 0.05.

Supplementary Table 1 Treatment of Patients. GP,gemcitabine plus cisplatin; PF, cisplatin plus 5-fluorouracil; TP, docetaxel plus cisplatin; TPF, docetaxel plus cisplatin plus 5-fluorouracil.
