# Supplementary figures and images for: A Nomogram to Predict Survival in Patients With Locoregional Recurrent Nasopharyngeal Carcinoma Receiving Comprehensive Treatment
Source: Front Oncol. 2022 Jun 16;12:892510. doi: 10.3389/fonc.2022.892510 (PMC9243306; doi:10.3389/fonc.2022.892510)

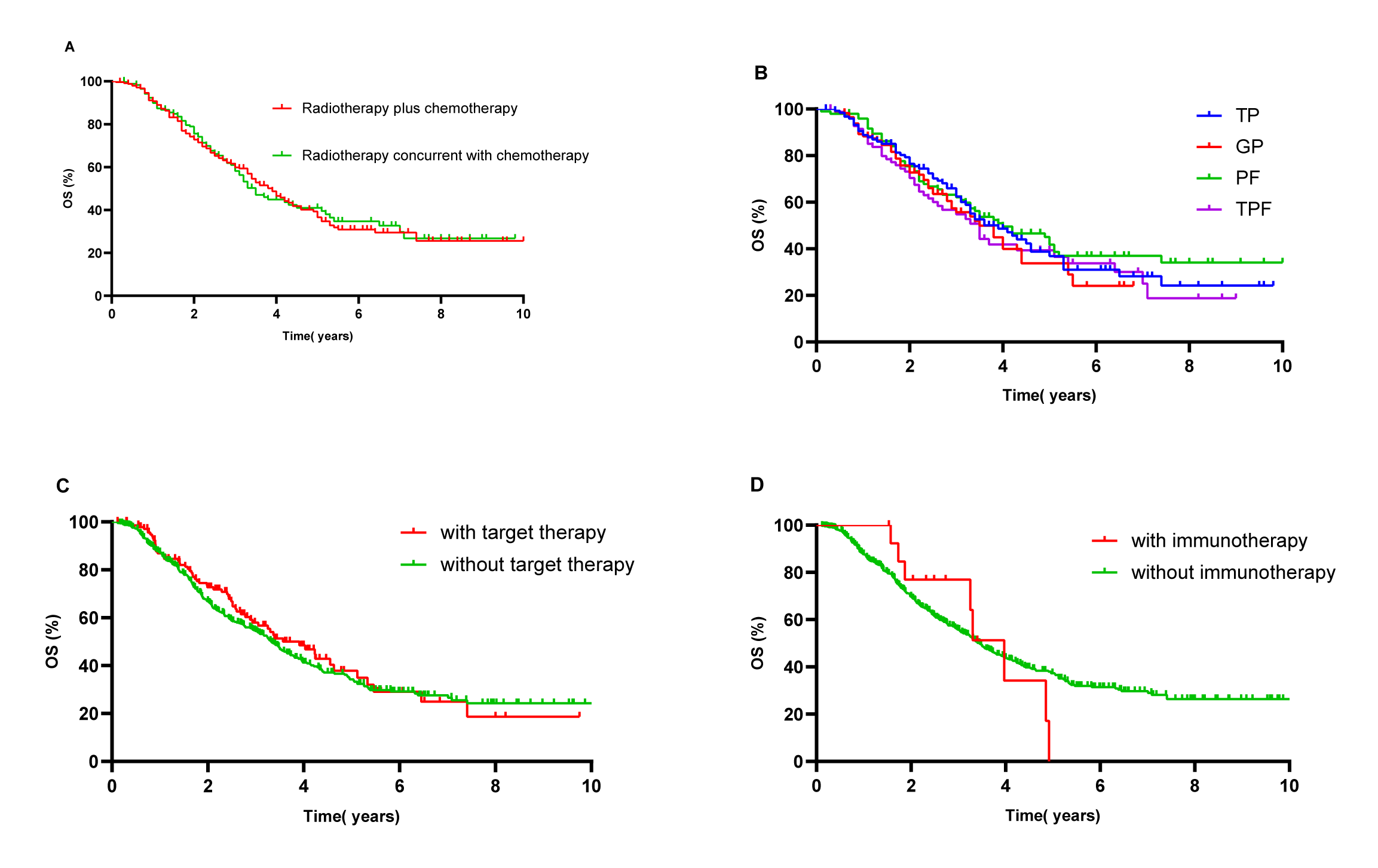

Supplement: Supplementary Figure 1 — Over survival in different treatment groups. (A) Difference in survival among populations with different radiotherapy conditions. Radiotherapy concurrent with chemotherapy vs. radiotherapy plus chemotherapy, p=0.910 (B) Difference in survival among populations with different chemotherapy regimens GP vs. TP, p=0.436; GP vs. TPF, p=0.905; GP vs. PF, p=0.331 (C) Difference in survival among populations with different targeted therapy conditions. received targeted therapy vs. without received targeted therapy, p=0.230; (D) Difference in survival among populations with different immunotherapy conditions. received immunotherapy vs. without received immunotherapy, p=0.196; 2-tailed α risk is 0.05. [file Image_1.tif]
