# Supplementary material for: A Nomogram to Predict Survival in Patients With Locoregional Recurrent Nasopharyngeal Carcinoma Receiving Comprehensive Treatment
Source: Front Oncol. 2022 Jun 16;12:892510. doi: 10.3389/fonc.2022.892510 (PMC9243306; doi:10.3389/fonc.2022.892510)
Supplement: Supplementary Table 1 — Treatment of Patients. GP,gemcitabine plus cisplatin; PF, cisplatin plus 5-fluorouracil; TP, docetaxel plus cisplatin; TPF, docetaxel plus cisplatin plus 5-fluorouracil. [file Table_1.docx]

## Table S1. Treatment of Patients.

|  | Low risk (N=202) | High risk (N=220) | Total (N=422) |
| --- | --- | --- | --- |
| Radiotherapy plus chemotherapy | 119 | 127 | 246 |
| Radiotherapy concurrent with chemotherapy | 83 | 93 | 176 |
| Chemotherapy regimen | | |  |
| GP | 57 | 61 | 118 |
| PF | 51 | 46 | 97 |
| TP | 54 | 69 | 123 |
| TPF | 40 | 44 | 84 |
| Targeted therapy | |  |  |
| Yes | 49 | 69 | 118 |
| No | 153 | 151 | 304 |
| Immunotherapy | |  |  |
| yes | 6 | 2 | 8 |
| no | 196 | 218 | 414 |

Abbreviations: GP,gemcitabine plus cisplatin ; PF, cisplatin plus 5-fluorouracil; TP, docetaxel plus cisplatin; TPF, docetaxel plus cisplatin plus 5-fluorouracil.
